# Privacy and Biometric Passports

**DOI:** 10.1100/tsw.2011.49

**Published:** 2011-03-01

**Authors:** Ioannis Vakalis

**Affiliations:** Institute for the Protection and Security of the Citizen, Joint Research Centre, Ispra, Italy

**Keywords:** electronic passport, privacy, biometric data, personal data, identification

## Abstract

This work deals with privacy implications and threats that can emerge with the large-scale use of electronic biometric documents, such the recently introduced electronic passport (e-Passport). A brief introduction to privacy and personal data protection is followed by a presentation of the technical characteristics of the e-Passport. The description includes the digital data structure, and the communication and reading mechanisms of the e-Passport, indicating the possible points and methods of attack.

## INTRODUCTION

The electronic passport (e-Passport) was introduced as the digital extension of the classic paper passport. The paper passport was, in its standardized form, the basic identification travel document for years. The electronic extension was decided upon as a security enhancement and to make identification more reliable for the authorities at the borders and for other state-based access controls.

Currently, passports are equipped with contactless smart card components, i.e., a chip and a radiofrequency identification (RFID) antenna. The chip contains biometric information and other biographical data permanently stored. A facial digital image is included; the same as is attached to the page together with other biographical data. From 2009, one fingerprint template has been added. Obviously, it is necessary to investigate which are the requirements for the protection of the e-Passport personal data as well as the exposure to risks while using the e-Passport.

The main problem with biometrics is that personal data directly referring to parts of the body of the subject, once lost or dispersed, are not replaceable. Also, the inclusion of the biometric data is obligatory for the new passport and the citizen does not have the option not to supply biometric information.

The biometric data are provided during the registration of the subject to the corresponding state authorities and the process is established by special law. The states themselves are responsible for the overall project of issuing, managing, and maintaining the system. For the latest extension of the e-Passport with the “extended access control” (EAC), this mechanism is enforced with electronic signatures and authentication keys that require a public key infrastructure (PKI).

The personal data protection, therefore, relies on the capability and the protection mechanisms of the administration (border police and other) during the life cycle of the e-Passport issuance, use, and withdrawal/destruction.

It should be underlined that apart from the risks for personal data loss during the daily use of e-Passport, additional threats exist that are targeted to the databases where the passport personal data of a citizen are kept as reference.

This document describes the important issues arising with the use of biometrics as means of identification and authentication, particularly with e-Passports, but also valid for future applications such as electronic identification (eId).

## PRIVACY

There have been a number of attempts to define privacy in a rigorous way, apart from the common perception shared by the public, which varies quite a bit among different cultures. Many aspects of privacy have been argued and described in numerous publications. At the European level, there is no legal definition of privacy, but it is described as one of the basic rights of the citizens of Europe (Directive 95/46/EC[1]). Basically, privacy is perceived in the context of personal data protection and the law applied refers to capturing, storing, and processing personal data (Article 8[2]).

Privacy for the average citizen can be summarized in a number of basic issues for which he/she perceives that action should be taken, for protection and control. So privacy is interpreted as the right to be left alone, to be anonymous, not to be tracked, and to protect personal information.

Privacy is not a rigid concept and there are differences in how it is perceived by individuals and groups of citizens. There are personal, national, and cultural differences among people about what privacy means and views may shift in time. Also, technical evolution affects the views on privacy since the ability to collect, process, and store data changes dramatically from year to year.

Large-scale biometric applications and other personal data collection projects have been initiated in recent years in reaction to security problems and threats that affect peoples' mobility. Numerous biometric databases have been established and are populated at the national level with different themes: illegal immigrants, visa holders, temporary workers, etc. on the basis of security protection.

Recommendation documents with certain principles, which should be adopted while designing and implementing a process or business model that includes personal data processing, have been published. According to the directives, any process that regards data "capturing, transmitting, manipulating, recording, storing or communicating", is considered subject to the data protection legislation.

Many European states, in response to the need for concrete steps for the application of the directives, published guidelines with issues and terms to be considered. The Dutch Personal Data Protection Directive[3] is a good example, focusing on issues like transparency, justification, legitimate grounds, and quality for the purpose.

Additional terminology was introduced in an effort to define privacy in concepts that can be interpreted in a technical context or even to become measurable. Some of the introduced terms are anonymity, unobservability, pseudonymity, and unlinkability[4].

Further effort was done to translate issues as listed above, in the form of rules applicable to real systems involving personal data and privacy aspects. Researchers devised rules such as proportionality, privacy by design, data minimization, and deployment of privacy-enhancing technologies (PETs)[3].

## PRIVACY-ENHANCING TECHNOLOGIES

PETs is a collection of information and communication technologies that strengthens the protection of an individuals' private life in an information system by preventing unnecessary or unlawful processing of personal data, more by offering tools and controls to enhance the citizens' control over their personal data. It complements the legal system that may not be sufficient in cases when personal data are disseminated in large geographic areas through computer and communication networks, and the processing of data encounters several jurisdictions.

The employment of PETs is expected to impact a variety of sectors and aspects of society and economy by promoting basic trust in provided services. Rather than witnessing the increase of privacy-invasive technologies, that technology becomes a medium to strengthen data protection and privacy of individuals. The use of appropriate technological measures is a substantial complement to legal means and should be an integral part in any efforts to achieve a sufficient level of privacy protection. PETs may include anonymization, pseudonyms, filters, PINs, and all related tools of information technologies.

## PRIVACY AND SECURITY

Although there is a general tendency among the public and specialists to put these two concepts in total opposition, they are not completely contradicting terms. There is a classic view that more security needs more information and, therefore, more personal data storing and processing and more surveillance, resulting in more privacy invasion. This is true to a certain extent, but can be handled better with regulations and technological means such as PETs. The security problem usually refers to certain security objectives and how they are achieved.

Depending on the case, it is suggested that all stakeholders open an early discussion among all interested parties. Societal consensus is possible by increasing trust, and using controls and checks for compliance with citizens’ rights and national constitutions.

Despite the efforts, the legislation, and the research for technical solutions, problems remain that are hard to resolve and are inherent to the issue of privacy. Among them, two are considered most important. The first is the linking of nonsensitive data (or anonymized data), becoming then sensitive data. The second is when personal data are transmitted to countries outside the EU jurisdiction that have different or no legislation regarding privacy and data protection. Security technologies and measures should be in line with the rights of the citizen.

Regarding the limits between the issues of privacy and security, the fine discerning lines could be defined in the following practices. Everyday threats or terrorism threats do not justify privacy infringements. Physically intimate technologies are unacceptable as they violate the citizens’ sense of private space. Finally, misuse of technology must be prevented and function creep is not acceptable.

Factors regarded as promoting the biometrics acceptability by the citizens are proportionality, legislation, strict control, and limitations on privacy compromising security technologies. National authorities and institutions should be sensitive to democratic demands for informative and open public debates that include broad involvement of relevant parties. Finally, it should always be required that privacy impact be analyzed before implementing projects or systems involving personal data.

Guidelines to be followed while thinking about privacy vs. security in real life should take into consideration that privacy and security should not be regarded as a zero sum game, and continuous evaluation and development is required.

The main bottleneck for establishing a system of controls and checks to ensure that entities using personal data comply with the legal condition is that such a system will be time consuming. To perform in-depth checks of legal compliance as well as technological-organizational measures requires knowledge of the audited system as well as a detailed roadmap of its functions. Applying only high-level checks, by either legal or technical experts, serious problems may go unnoticed. A broad spectrum of experts is required, as well as establishing a data-controller role in major information computer technology (ICT) systems.

There are many difficulties on how to protect privacy from infiltration attacks and consequent manipulation of evidence. These are problems strictly related to security of the ICT infrastructure. Possible avalanche side effects can result in networks with highly interconnected computers influencing other computer functionalities. Best practices should be provided that could reach drastic actions such as controlled termination and, at the same time, guidelines to avoid technical practices that cultivate ICT vulnerabilities.

## BIOMETRICS

In the last 20 years, biometrics have emerged as one of the primary methods to identify persons. In the past, they were used in the crime scene milieu for identification mainly. Certain properties of biometrics regarding the identified subject attracted the attention of specialists in the security field. With respect to identification or authentication methods (something you know, have, are), biometrics falls into the category "something you are". Biometrics are intrinsic properties of the physical body of the subject; they are not possible to share and are difficult to duplicate. Biometrics are impossible to be lost, only in the case of an accident. Biometrics are by far the most effective means for personal identification, promoting security, interoperability, availability, and efficiency in border- and other state-induced access controls.

There are many paradigms and functioning models for biometrics, the most illustrative, but arguable, is that of the "anchor"[5]. More precisely, stored information depicting parts of the human body creates an "anchor" to other personal data so that these data are strongly related to the physical body (Fig. 1).

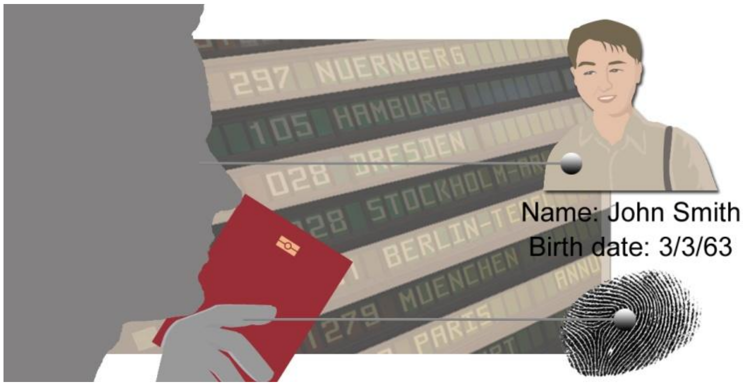

**FIGURE 1.** Biometrics function as anchors to the physical body of a person.

This strong relationship of biometrics with the physical body that makes them accurate and reliable identifiers also has a negative effect in that they can be used as a tool to compromise privacy. Particularly, using and processing biometric data in digital form allows tracking, profiling, identification, and other forms of privacy attack.

## REQUIREMENTS FOR BIOMETRICS IN IDENTIFICATION DOCUMENTS

There is no legal definition of what constitutes biometric data in European law. Instead, there is a legal definition of what constitutes personal data. The existing legislation[1] assumes "personal data" to be any information relating to an identified or identifiable natural person. In this case, identification refers to all available ways to identify that person, e.g., by reference to an identification number or to one or more attributes specific to his/her physical, physiological, mental, economic, cultural, or social identity. The basic characteristic of biometric data is that they are strong identifiers manifesting the "anchor" property. On the basis of this fact, it is generally accepted that biometric data follow the legislation concerning the use of personal data. In a number of legislative acts, European law suggests the reduction of the processing of personal data to the unavoidable extent, maintaining the highest transparency possible, and institutional and individual control of processing personal data as efficiently as possible.

A few years ago, the only biometric part of identification documents and passports as well was the picture used as a tag to connect the information in the passport to the physical person. Identification took place by inspection (manually) in front of an official who judged if the picture corresponded to the physical person.

The first generation of e-Passports was provided with the capability to include electronic data stored within a chip and a digital copy of the facial photo as biometric data. The next generation of European e-Passports also included a fingerprint template.

Establishing and maintaining the infrastructure for the use of the e-Passport, including biometric registrations and matching mechanisms, is a costly operation since the targets are the populations of whole countries.

In Fig. 2, the process diagram for a generic biometric matching is presented, as well the points where interference on the process by impostors or hackers may happen[6]. The attacks have a different technical basis for every point:

1. Spoofing
2. Change the captured data or image
3. Override feature extraction algorithm
4. Change the extracted features
5. Override matcher
6. Change the stored template
7. Change the template sent to matcher
8. Override the decision by the matcher

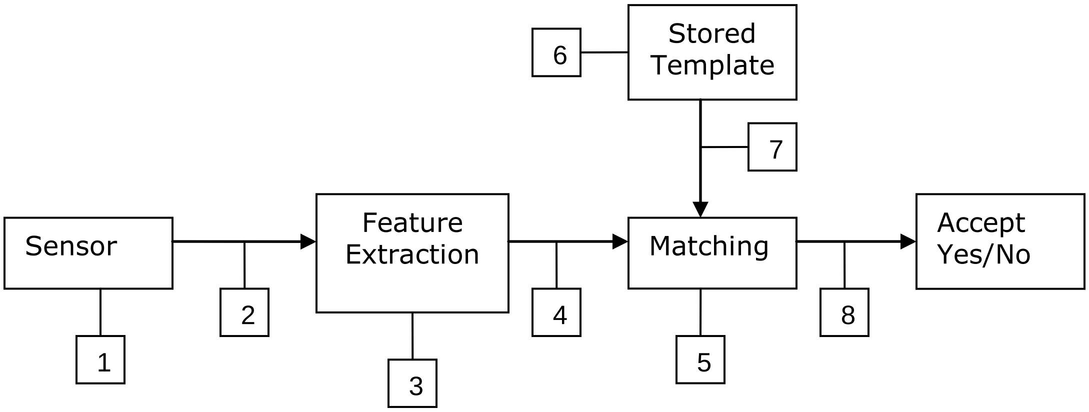

**FIGURE 2.** The biometric identification stages[6].

Attacking the biometric matching process at any point is considered a privacy threat in the sense that the attacker can access information that includes personal and other sensitive data. Obtaining biometric data directly in the form of an image or template is also a direct privacy threat for biometric systems. Tracking and profiling are the possible intentions of attacks; for other actions, further processing of the data may be needed. Point 4 in Fig. 2 and the storage space are the relative attack points.

The performance of biometrics depends on many parameters within a system, including the matching algorithm. The basic parameter is the statistical threshold that defines the acceptance score. Important performance indices for a biometric system are the false acceptance rate (FAR) and the false rejection rate (FRR)[6]. The FAR can only be measured in laboratory conditions and not in real-life applications.

The biometric e-Passport belongs to large-scale biometric applications that have been launched for security reasons. Many of them regard border crossings and immigration, such as the U.S. visitors' visa and the EU–Schengen biometric visa. Large-scale biometric applications bring the contemporary citizen into a new realm with effects not yet obvious. It will take some time to study the effects of biometric systems of such scale.

## THE NEW E-PASSPORT AND ITS BIOMETRIC DATA

The evolution of cryptographic tools, networking methods, and the related information technology (e.g., smart cards) gave a thrust to the application of electronic credentials and certificates in various domains such as e-government, e-banking, etc. The use of biometric certificates and other means of identity management in services and everyday life are constantly increasing and applications reach many citizens. The transformation of personal information into electronic data raises new challenges as well as threats and risks, particularly in large-scale applications. A few years ago, there was a tendency in the airline and airport business to use such identification methods in order to allow frequent flyers with a trusted profile to access faster security controls. The airline industry sector was and still is considered one of the most threatened globally, and that is why new methods and tools are constantly sought in order to enhance security and usability at the same time. These methods contain the latest technology, including contactless smart cards, with encrypted biometric information and other credentials. These processes can also be privacy driven if there is control in the use and storage of personal data, including biometric. A domain of research and development emerged in response to privacy and identity management (PIM) challenges and, at the same time, offered opportunities for designing architecture components for device-centric applications that process personal data. A challenging characteristic of device-centric applications is the implicit nature of interactions between the user and applications by means of devices acting as independent agents. These devices (smart card, RFID) have limited power and processing capabilities and, hence, we can shape their behavior according to the needs of the targeted applications; however, at the same time, they do not offer much sophistication.

It was natural for specialists searching for ways to enhance the security features of passports to focus their attention on this technology. The electronic chip was introduced to the normal paper passport in an effort to make the passports more reliable and harder to forge or duplicate. Although the International Civil Aviation Organization (ICAO) was working in the area of machine-readable travel documents (MRTD) since 1968 and established the standard for the machine-readable zone (MRZ) in 1980, some time passed before it was possible to establish a standard for the e-Passport. The basic problem was the technological limits of the integrated circuit (IC) chip memory capacities that could not store enough information, e.g., the size of a picture. As the chip technology evolved and made available contactless IC chips with enough memory (32 kB), in 2004 the ICAO published the first version of the e-Passport standard[7].

According to the ICAO standard, the new e-Passport contains within the IC chip most of the information included on the printed page (Fig. 3). This includes the normal biographic data, but also an electronic form of the facial photo. The facial photo constitutes biometric data and therefore all the issues regarding biometrics are relative. In 2009, the EU adopted a standard for an extended biometric passport to include a fingerprint and some additional enhanced security controls (Fig. 4).

The relative ICAO document[7] describes the organization of the stored information in the e-Passport chip, which is called logical data structure (LDS). As shown in Fig. 3, the information is organized in data groups (DG) and there are three optional data groups not appearing in the figure: Automated Border Clearance (DG17), Electronic Visas (DG18), and Travel Records (DG19). From all the data groups, only DG1 (biographic data) and DG2 (photo) are mandatory. The DG15 includes the public key of the chip.

The access to the photo, which is in JPEG 2000, and the fingerprint template is controlled by cryptographic keys. The adoption of the e-Passport was done with legislation at the EU level and the capture and storage of the biometric information is covered by law.

| | | | |
|---|---|---|---|
| Detail(s) Recorded In MRZ | DG1 | | Document Type |
| | | | Issuing State or Organization |
| | | | Name (of the Holder) |
| | | | Document Number |
| | | | Check Digit – Doc Number |
| | | | Nationality |
| | | | Date of Birth |
| | | | Check Digit –DOB |
| | | | Sex |
| | | | Date of Expiry or Valid Until Date |
| | | | Check Digit – DOE/VUD |
| | | | Optional Data |
| | | | Check Digit - Optional Data Field |
| | | | Composite Check Digit |
| Encoded Identification Feature(s) | Global Interchange Feat | DG2 | Encoded Face |
| | Additional Feature(s) | DG3 | Encoded Finger(s) |
| | | DG4 | Encoded Eye(s) |
| Displayed Identification Feature(s) | DG5 | | Displayed Portrait |
| | DG6 | | Reserved for Future Use |
| | DG7 | | Displayed Signature or Usual Mark |
| Encoded Security Feature(s) | DG8 | | Data Feature (s) |
| | DG9 | | Structure Feature (s) |
| | DG10 | | Substance Feature (s) |
| | DG11 | | Additional Personal Detail(s) |
| | DG12 | | Additional Document Detail(s) |
| | DG13 | | Optional Detail(s) |
| | DG14 | | Reserved for Future Use |
| | DG15 | | Active Authentication Public Key Info |
| | DG16 | | Person(s) to notify |

**FIGURE 3.** The data structure stored in the IC chip of the e-Passport[8].

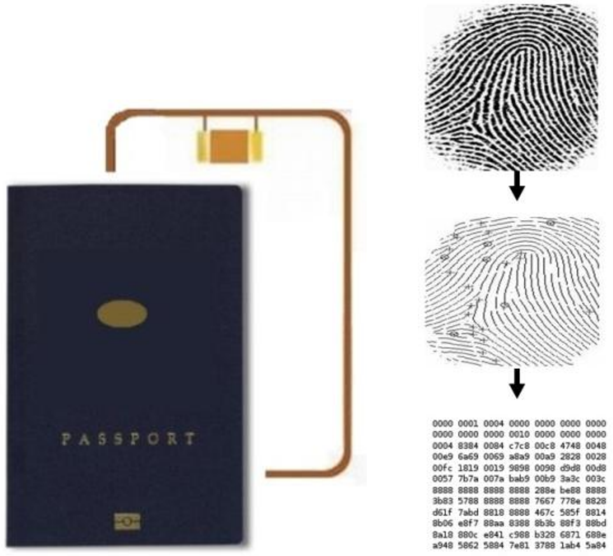

**FIGURE 4.** The new EU e-Passport includes a fingerprint template.

## THE RFID MECHANISM OF THE e-PASSPORT

A basic functional characteristic of the new e-Passports is the RFID component, which allows its use as a contactless smart card. The chip communicates with the reader/scanner through an RFID antenna that is usually embedded in the cover page of the passport. There are a number of reasons for using the RFID contactless mechanism, among them to reduce the risk of direct attacks to the otherwise metal contacts. With contactless technology, usability problems and the wearing out of contacts can be avoided. The ISO standard 14443 is used. There is a 32-bit number used for communication collision avoidance when there are more passports present in the proximity of the reader. To prevent tracing of the passport, the emitted number can be random, although some countries have constant numbers. The ISO 14443 specifies that the number should start with byte 08 to differentiate from other emitted sequences. Some countries have decided not to follow this rule, but by doing so, the reading of the number reveals the origin of the passport. In this case, uniformly applying the standard would reduce risks. The fact that the random number is generated with 08 in the beginning of the bit string poses a vulnerability risk as it gives the information that the contactless chip is possibly an e-Passport and can be targeted for unauthorized reading. Some countries have introduced metal shielding into the covers of their passports, thus creating a Faraday cage (i.e., to block external electromagnetic fields) in order to allow reading only when the owner opens the document, thus giving his consent.

In RFID-related literature, there are references on how its misuse could infringe on the individuals' right to privacy and protection of personal data possibly transmitted by RFID antennae. In that case, RFID applications fall within the scope of the data protection directive, and the e-Passport is one such application.

A recommendation document[9] has been published by the European Commission on the implementation of privacy, data protection, and information security principles in applications supported by RFID. The purpose of the recommendation is to provide guidance by identifying principles in relation to RFID use that would seek to ensure maximizing benefits of RFID use without compromising the right to integrity, privacy, and data protection of the individual in a democratic society.

## BASIC ACCESS CONTROL (BAC)

The ICAO standard requires that the BAC is implemented for each e-Passport to allow the communication between the IC chip and the device that reads the chip. Actually, the BAC is used for access control and key agreement between the two devices.

Initially the reader scans the MRZ of the passport (the zone of two rows and 44 characters in the lower part of the main passport page). The MRZ includes the name of the passport holder, the country number, date of birth, and the expiration date. The reader optically scans this zone and then records the information used as input to the BAC algorithm in order to create a 2-key triple DES[10] and authenticates the reader. The BAC actually makes sure that the reader has scanned the MRZ in order to prevent unauthorized access and passive eavesdropping of the information on the IC chip. The basic idea behind this is that someone opens his/her passport so that the MRZ is scanned and knowingly allows the authorities to read his/her passport.

The key agreement in detail works as follows. The first step is to use MRZ information in the SHA1 algorithm[10] to open secure messaging. In a second step, secure messaging is tested mutually between the reader and the IC chip. Then, two random numbers are exchanged and the XOR[10] of the two numbers is used as the seed to derive a session-dependent symmetric key.

There are some problems with this method since the primitives for the key construction are one-way (from the passport) and they are not considered secure enough for the key agreement protocol. The information on the MRZ is low entropy, and the chip could be read with online and offline brute force attack, making the whole scheme vulnerable. A passive listener between the reader and the passport could run an offline exhaustive search. Online attacks have been demonstrated within distances of 1.5 and 4 m.

There are indications for experiments up to a distance of 10 m. Therefore, an obvious tactic would be an offline attack to deduce the key and then, with the initial key in hand, an online attack to capture the information stored in the chip.

Apparently from the security point of view, an authenticated key exchange protocol system would be better in this case. That is why the EU introduced the passport with the EAC to protect the personal data in the IC chip or the passport.

### Passive Authentication

The ICAO standard defines the use of the PKI and digital signature to authenticate the digital information in the chip and this mechanism is called passive authentication. This is mandatory for the ICAO standard.

In addition to the data groups stored in the passport chip, another file exists called the security object for the document (SOD). The SOD includes the list of the hashes[10] to all data groups and a signature to this list by the issuer document signer (DS). The certificate of the DS public key may also be included. Otherwise, this certificate can be obtained by the ICAO public-key directory. It is a responsibility of each country to organize its own PKI mechanism and to use diplomatic routes to send to all other countries a self-signed certificate to the root. There is also a revocation process that is done periodically. The signature algorithms that sign the DS certificate and the SOD file are SHA[10] with RSA and RSA[10] in passive authentication.

### Active Authentication

In addition, an active authentication (AA) mechanism is foreseen to authenticate the IC chip to the reader also using the public key (the same PKI as for passive authentication) mechanism, but there is a challenge response to prove that the chip holds the corresponding secret key.

The AA is not mandatory and is implemented by few countries. We can see in the following that the EAC covers most of the aspects of AA. When AA is implemented, the DG15 holds the key.

## THE EUROPEAN EAC

The EAC mechanism was not standardized by the ICAO. It was left optional and requires a bilateral agreement between two or more countries for the exchange of cryptographic keys. With all the concerns that emerged with BAC, the EU took the initiative to launch the European EAC as a harder access control mechanism in 2006. The European EAC is implemented and applies to all the countries in the Schengen area. When reading a passport, EAC comes after the passport passes the BAC, which is mandatory. It consists of three authentication submechanisms: chip authentication, terminal authentication, and passive authentication (Fig. 5). The chip authentication regards the authenticity of the electronic part of the passport. The terminal authentication refers to the passport readers installed at borders or airports. Finally, the passive authentication mandated by the ICAO corresponding standard is included as part of the EAC.

The EU passport standard requires an additional biometric, i.e., one fingerprint template implemented after 2009 (Fig. 4). Actually, EAC was introduced to protect more sensitive personal data like the fingerprint (or other biometric in the future). The distinction to BAC is that biographical data and the facial photo are considered to be less sensitive. The ideal would be to include all the data in EAC in the future.

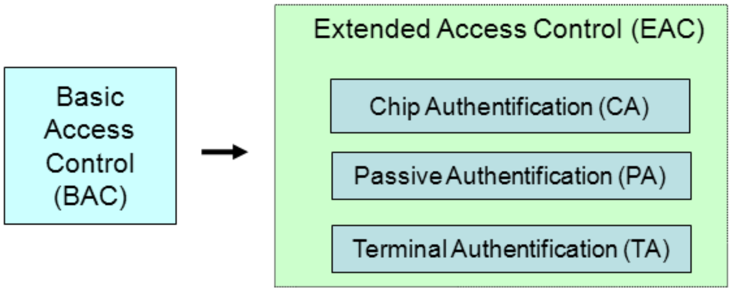

**FIGURE 5.** BAC and EAC for the new EU e-Passport.

Chip authentication consists of a Diffie-Hellman (DH) protocol[10]. Therefore, passive eavesdropping is not possible. The chip uses a static DH public key and the reader uses a 1-day key. The chip public key is authenticated (passively) by SOD to the reader so the key agreement is semi-authenticated. In the next step, a key is produced for secure messaging.

Terminal authentication is the mechanism for the reader (scanner) authentication based on a PKI. The terminal gives its digital certificate to the IC chip, which in turn sends back a random challenge. The terminal signs the challenge with its 1-day DH key. The IC chip then checks the signature.

The PKI for the readers is separate from that of the passport and requires a certificate for each authorized reader. All involved countries should have another similar PKI mechanism for readers. In contrast with the passport keys, which expire with the passport itself, the readers have shorter-term certificates. This is to protect passport scanning from stolen readers.

The basic claim behind the EAC is that the terminals do not have the privacy problems like that of the BAC with the semantic attack vulnerability. EAC is an example of PET application.

## SECURITY AND PRIVACY ISSUES OF THE e-PASSPORT

As pointed out in previous paragraphs, the old passport included personal and biometric (photo) data and, in this manner, there was a data loss risk associated with its use. The data risk was restricted to the scope of the authorities. In addition, the classic passport was used as an identification token, for example, in hotels or banks where data could also be retained (e.g., photocopy).

The e-Passports include electronic personal information (plus biometrics) that is very easy to read, store, and transmit. This technical characteristic offers an additional method to access and store personal data. The RFID communication method may pose an additional problem for privacy because tracking is possible; even if the 32-bit emitted numbers are random, the first two digits (08) always spot the passport.

RFID metal shields can protect this problem, but they ring on metal detectors.

As was described in the relative paragraph for the BAC mechanism, it is not based on public key cryptography and this allows online and offline brute force attacks because of the low entropy of the MRZ information. In addition, passive eavesdropping can then lead to an offline brute force attack.

The new passport poses a privacy threat in comparison with those of the past since a copy of the paper passport was not evidence of the presence or the involvement of a person in a specific location or a transaction[13]. The passports now, together with the digital information, are a proof since a digital copy of the LDS or the SOD certified can be obtained and retained. Passports are shown in hotels and duty free shops where an employee could create copies of the digital information, which then becomes strong evidence.

Automatic identification is driving the latest innovative solutions in the area of border controls. In the first step, the e-Passport is scanned by the reader and controlled for the validity of the digital data. The facial picture, which should be a high-quality image (JPEG 2000) and stored in DG2, is kept momentarily and used for automatic face recognition, which is performed in the second step. The facial matching requires that a new facial image of the subject be taken every time. In such a system, it is obvious that data protection methods and the security should be of high level. Any loss or leakage of data can facilitate identity- and privacy-threatening activities.

For the present time, the e-Passport is not being used in other identification procedures, except in border control, at least as far as it concerns the digital part. The risks can be augmented if automated identification is applied in other sectors, e.g., banking based on e-Passports. Another risk with automated identification is that a low-quality clone of a genuine passport would pass easily, particularly if the facial image had a resemblance.

The embedded IC chip can become a target of attack and if it gets corrupted, the static DH key may then leak and the communication can be decrypted.

EAC makes it hard to attack the biometric data in the passport chip, but some of the content of the LDS can be recovered; in the case that after passing from the BAC, the SOD file is released. Then it is easy when the passport owner is matching his fingerprint against the stored template, having a close to template sketch to be able to obtain the original, by brute force.

Other information stored in the chip can be recovered, having the digest from the SOD. There are methods to reduce the risk of this kind of attack.

As said in previous paragraphs, the transfer of the certificates or one of the keys is a major security and privacy threat. Certain type of threats, known as Mafia attacks or middleman attacks, take place when a third agent interferes between the passport holder and the reader, functioning as a verifier[12].

There are more advanced protocols that can be applied with a PKI to strengthen the existing system. Universal Designated Verifier Signature (UVDS) and similar schemes, such as UVDS Proof with Protocol, have been proposed to avoid the transferability of certificates and signatures[11].

## CONCLUSIONS

Up to a few years ago, the only biometric part of a passport was the picture, which was used as a tag to connect the information in the passport to the physical person, in his presence, mainly at border crossings of a country. Identification was done manually in front of a border official who judged if the picture corresponded to the physical person. In addition to its main function, the passport was used to identify persons in hotels, banks, and a number of other activities.

The first generation of e-Passports provided the possibility to store digital personal data, which are certified and signed. This constitutes a paradigm shift and the access to passport data has a different effect; both authorities personnel and the public should be informed about the effect of this change and the personal data protection practices should be applied on the total business model of the identification processes.

A conclusion resulting from the above analysis is that a good tactic will be to put limits on who and how the passport can be read, since “reading” means:

- Access to a chip in which are placed biometric data; sensitive personal information.
- Read the MRZ; you receive a copy of digitalized information that is resident on the document and readable with the naked eye.
- Access the chip with the BAC protocol; means access to digital form of the data inside the chip, the same as on the printed part of the document, and the photograph too.

Both authorities and the public should be informed about the changes with respect to the past.

- Authorities are allowed to access data of an e-Passport; some of them are not visible on the document, but stored in the chip.

- The e-Passport personal data have a great value, particularly for the owner; all must be done to protect them and to legally defend all attempts to corrupt or steal them.

It is important to understand that the PKI system can be compromised, with key leakage, and the side effects by far exceed the loss of one passport and the biometrics with it.

- State PKI must be built with high security standards, quick response time to incidents, and interoperability.
- The access control systems to the various databases of law enforcement authorities have to be upgraded in order to ensure that data are not at risk when accessing them.

The e-Passport also offers the opportunity to use automatic and unattended border controls, which is a recent trend in border crossing. The e-Passport has been proposed as an identification token for network-based processes[14].

## DISCLAIMER

The opinions and the views expressed in this work by the author are not necessarily those of the Joint Research Center and the European Commission.
